# Integrated Lipidomics and Network Pharmacology Reveal the AMPK-Mediated Therapeutic Mechanism of 3,3′-Diindolylmethane in Hepatic Lipid Metabolism

**DOI:** 10.3390/antiox14091093

**Published:** 2025-09-07

**Authors:** Xudong Li, Yunfeng Lin, Ruomei Niu, Siyuan Chen, Jingyun Pan, Yuquan Zhong, Junqiang Du, Qiuxia Dong, Hongfeng Zhang, Heng Fang, Huiyang Zhu, Wei Zhu

**Affiliations:** 1 Institute of Public Health, Guangzhou Center for Disease Control and Prevention, Guangzhou Medical University, Guangzhou 510180, China; lxdnutrition@gzhmu.edu.cn (X.L.); 2112241052@stu.gdpu.edu.cn (Y.L.); 2023211473@stu.gzhmu.edu.cn (R.N.); chensiyuan@stu.gzhmu.edu.cn (S.C.); 2023211452@stu.gzhmu.edu.cn (J.P.); 2022211438@stu.gzhmu.edu.cn (Y.Z.); 2024211617@stu.gzhmu.edu.cn (J.D.); 2024211621@stu.gzhmu.edu.cn (Q.D.); fangh36@mail2.sysu.edu.cn (H.F.); 2 Department of Scientific Research, Guangzhou Center for Disease Control and Prevention, Guangzhou 510440, China; gzcdc_guoyh@gz.gov.cn (H.Z.); gzcdc_zhuhy@gz.gov.cn (H.Z.)

**Keywords:** 3,3′-diindolylmethane, MASLD, AMPK, lipid metabolism

## Abstract

Dysregulation of hepatic lipid metabolism constitutes a central mechanism in the pathogenesis of metabolic dysfunction-associated steatotic liver disease (MASLD). 3,3′-Diindolylmethane (DIM), a bioactive compound abundant in dietary Brassica vegetables, exhibited protective effects on hepatocellular carcinoma and metabolic/inflammatory pathologies. Nevertheless, the effects of DIM on hepatic lipid metabolism and its underlying mechanisms remain unclear. Administration of DIM (50 mg/kg bw/day) prevented oxidative stress and hepatic lipid deposition in both high-fat diet (HFD)-fed wild-type (WT) and ob/ob mice. Lipidomics revealed that DIM diminished the lipogenesis and reshaped the hepatic lipid profile. Network pharmacology analysis identified the AMPK signaling pathway as the underlying mechanistic target for DIM in treating MASLD. In both HepG2 cells and mouse primary hepatocytes (MPH), DIM attenuated palmitic acid (PA)-induced cellular lipid accumulation, ROS generation, and reduction in oxygen consumption rate (OCR). These protective effects of DIM were diminished by co-treatment with Compound C (CC), a specific AMPK inhibitor. DIM administration enhanced AMPKα phosphorylation in vivo (WT/ob/ob mice) and in vitro (HepG2/MPH), concomitant with PPARα upregulation and SREBP1/ACC1 downregulation. CC abolished all DIM-induced molecular changes in vitro. Collectively, DIM alleviates hepatic lipid accumulation and oxidative stress in MASLD models through AMPK activation, subsequently modulating PPARα and SREBP1/ACC1 pathways.

## 1. Introduction

Metabolic dysfunction-associated steatotic liver disease (MASLD) is a chronic liver disorder whose initial stage is characterized by hepatic steatosis [1]. Physiological hepatic lipid regulation involves balanced free fatty acids (FFA) uptake, de novo lipogenesis (DNL)-derived triglyceride synthesis, β-oxidation, and very low-density lipoprotein (VLDL)-mediated triglyceride (TG) export—collectively maintaining hepatic fat content below 5% in healthy individuals. However, dysregulation of this homeostatic triad, characterized by enhanced DNL, diminished β-oxidation, and reduced TG export, impairs hepatic lipid equilibrium. This imbalance drives excessive intrahepatic lipid accumulation, initiating MASLD [1].

As dominant environmental triggers, high-fat diet (HFD) dysregulate hepatic lipid fluxes and promote lipotoxicity, accelerating MASLD progression from steatosis to steatohepatitis [2]. HFD stimulated adipose tissue lipolysis, leading to an increased flux of FFA to the liver and consequent hepatic lipid accumulation [3]. Hepatic lipid accumulation induces oxidative stress, resulting in hepatocellular damage and subsequent exacerbation of inflammation and fibrosis in MASLD [4].

3,3′-Diindolylmethane (DIM), a bioactive lipophilic compound abundant in dietary Brassica vegetables (e.g., kale, broccoli, cauliflower), forms endogenously from indole precursors. DIM exhibits promising chemopreventive and antitumor activity in human trials, supporting its therapeutic potential against diverse cancers [5,6]. For hepatocellular carcinoma, DIM concurrently targets oncogenic pathways by reversing epithelial–mesenchymal transition (EMT), ameliorating oxidative stress, curbing cellular proliferation, inducing apoptosis, and blocking metastasis [7]. Beyond oncology, DIM exhibits beneficial activity in metabolic/inflammatory pathologies such as cardiovascular disease [8], rheumatoid arthritis [9], inflammatory bowel disease [10], and obesity [11]. Pharmacokinetic analysis demonstrated peak DIM concentrations in the liver following oral administration [12]. Despite DIM established metabolic effects, its impact on hepatic lipid metabolism and underlying mechanisms remained unknown.

In this study, we examined how DIM modulates hepatic lipid metabolism using an integrated approach combining in vivo and in vitro studies, lipidomics, and network pharmacology analysis. This study provides significant insights into the mechanism by which DIM improved MASLD.

## 2. Materials and Methods

### 2.1. Animals

All animal procedures strictly adhered to the Animal Ethics Procedures and Guidelines of the People’s Republic of China and received formal approval from the Animal Ethics Committee of the Guangzhou Center for Disease Control and Prevention (approval number: No.2023055). Eight-week-old male C57BL/6J wild-type (WT) and ob/ob mice were obtained from the Guangdong Provincial Laboratory Animal Center (Guangzhou, China). Animals were maintained under specific pathogen-free (SPF) conditions at 22 ± 2 °C with 30–60% humidity and a controlled 12 h light/dark cycle.

### 2.2. DIM Treatment

DIM (C_17_H_14_N_2_; MW: 246.31; purity ≥ 98.0%; Sigma-Aldrich, St. Louis, MO, USA) was dissolved in 0.1% sodium carboxymethyl cellulose (CMC-Na) to yield a 40 mg/mL solution (400 mg in 10 mL). Following a 2-week acclimatization period on a normal chow diet (NCD; 10% kcal fat; D12450J, Research Diets Inc., New Brunswick, NJ, USA), all 8-week-old male mice were randomly assigned to receive either NCD or a high-fat diet (HFD; 60% kcal fat; D12492, Research Diets Inc.). Concurrently, mice received daily oral gavage of either 50 mg DIM/kg body weight/day (human equivalent dose: 243.24 mg/day for a 60 kg individual) or an equivalent volume of CMC-Na vehicle. In mice, DIM can be gavage at 250 mg/kg bw with no toxicity and wide tissue distribution [13]. In preclinical studies, doses under 300 mg (using standard body surface area conversion [14], the human-equivalent dose (based on a 60 kg body weight) translates to a mouse dose of 61.67 mg/kg bw/day) were not associated with adverse events in either healthy subjects or patients with castrate-resistant, non-metastatic prostate cancer [15,16].

Briefly, WT mice were randomly assigned to one of the following three groups (*n* = 10/group): (1) NCD + vehicle; (2) HFD + vehicle; (3) HFD + DIM. ob/ob mice were randomly assigned to one of the following two groups (n = 8/group): (1) HFD + vehicle; (2) HFD + DIM. Body weight was monitored weekly. After 16 weeks (WT mice) or 10 weeks (ob/ob mice), animals underwent a 24 h fast prior to euthanasia via sodium pentobarbital overdose (1%).

### 2.3. Assay of Biochemical Parameters

Biochemical parameters were assessed as previously described [17]. Briefly, liver free fatty acids (FFA), TG, and total cholesterol (TC) (Nanjing Jiancheng Bioengineering Institute, Nanjing, China) were measured in the supernatant obtained from liver tissue homogenized in 95% ethanol. Hepatic reactive oxygen species (ROS) levels were measured using dihydroethidium (DHE) fluorescence [18]. Malondialdehyde (MDA) levels were quantified via thiobarbituric acid reactive substances (TBARS) assay. Superoxide dismutase (SOD) activity was determined by monitoring the inhibition of tetrazolium salt (e.g., WST-1) reduction to formazan by superoxide radicals generated in a xanthine/xanthine oxidase system [19]. Catalase (CAT) activity was assessed by measuring the decomposition rate of hydrogen peroxide (H_2_O_2_) [20]. Levels of ROS (Applygen Technologies Inc., Beijing, China), MDA, CAT, and SOD (Solarbio Science & Technology Co., Beijing, China) were measured in PBS-homogenized liver tissue. Serum levels of TG, TC, low density lipoprotein-cholesterol (LDL-c), high density lipoprotein-cholesterol (HDL-c), FFA, alanine aminotransferase (ALT), and aspartate aminotransferase (AST) were quantified using commercial assay kits (Nanjing Jiancheng Bioengineering Institute, Nanjing, China).

### 2.4. Histological Analysis

Liver tissues were fixed in 4% paraformaldehyde. Hematoxylin and eosin (H&E) staining and Oil Red O staining were performed by Servicebio (Wuhan, China). Digital images were acquired using an Axio Examiner light microscope (Carl Zeiss AG, Oberkochen, Germany). Hepatic TG content was quantified through morphometric analysis of Oil Red O-stained sections using Image-Pro Plus 6.0 software (Media Cybernetics, Rockville, MD, USA).

### 2.5. Western Blot

Western blot analysis was performed as previously described [17]. Briefly, protein lysates (50 µg per sample) were separated by SDS-PAGE and transferred onto 0.45 µm polyvinylidene difluoride (PVDF) membranes. The membranes were blocked with either 5% non-fat milk or 5% bovine serum albumin (BSA) in TBST, chosen according to the specific primary antibody requirements (BSA for phospho-specific antibodies). Following blocking, membranes were incubated overnight at 4 °C with primary antibodies (Table S1). Subsequently, species-appropriate horseradish peroxidase (HRP)-conjugated secondary antibodies were applied for 2 h at room temperature. β-Actin was used as the loading control. Protein band intensities were quantified using ImageJ software, version 1.48 (National Institutes of Health, Bethesda, MD, USA).

### 2.6. Mouse Primary Hepatocytes Isolation

Mouse primary hepatocytes (MPH) were isolated from 8-week-old male wild-type (WT) mice maintained on NCD. Following anesthesia, livers were perfused in situ with collagenase type II (Gibco, Carlsbad, CA, USA). Hepatocytes were gently resuspended in Williams’ E Medium supplemented with 10% (*v*/*v*) fetal bovine serum (FBS) and 1% (*v*/*v*) penicillin-streptomycin (10,000 U/mL penicillin, 10,000 mg/mL streptomycin; Gibco). Cells were seeded at 1.5 × 10^6^ cells per 35 mm culture dish and maintained at 37 °C in a humidified 5% CO_2_ atmosphere.

### 2.7. Culture of HepG2 Cell

HepG2 cells were purchased from American Type Culture Collection (Manassas, VA, USA) and cultured in DMEM containing 10% (*v*/*v*) FBS (Sijiqing, Hangzhou, China). The culture medium was changed every two days.

### 2.8. Cell Treatment

Sodium palmitate (PA) was dissolved in 20% (*w*/*v*) BSA solution at 55 °C to generate a 10 mM stock solution, with gentle vortexing until clear. DIM (C_17_H_14_N_2_; MW: 246.31; purity ≥ 98.0%; Sigma-Aldrich, St. Louis, MO, USA) and Compound C (CC, Dorsomorphin; Abcam, Cambridge, UK) were separately dissolved in dimethyl sulfoxide (DMSO) to prepare 10 mM stock solutions. Cells were treated with PA ± DIM for 24 h. For adenosine monophosphate-activated protein kinase (AMPK) inhibition studies, cells were pre-treated with 10 µM CC for 30 min prior to PA/DIM exposure.

### 2.9. Cell Viability Assay

Viability was assessed using CCK-8 (Dojindo Laboratories, Rockville, MD, USA) measuring WST-8 reduction. In total, 1 × 10^4^ MPH/well or 2 × 10^4^ HepG2 cells/well were seeded in 96-well plates, adhered overnight, and treated with PA or DIM at different concentrations for 24 h. After treatment, 10 μL CCK-8 was added, followed by 2 h incubation (37 °C, 5% CO_2_). Absorbance (450 nm) was normalized to untreated controls.

### 2.10. Seahorse Assay

Mitochondrial oxidative phosphorylation in MPH was evaluated using an XF24 extracellular flux analyzer (Seahorse Bioscience, North Billerica, MA, USA). Cells were plated in XF24 culture plates and cultured to ~90% confluency. Prior to the assay, culture medium was replaced with assay-specific medium and incubated at 37 °C for 1 h. Following baseline oxygen consumption rate (OCR) measurements, specific compounds were sequentially injected into individual wells. OCR (pmol/min) was measured after each injection. All values were normalized to total cellular protein content quantified from cells harvested immediately post-assay.

### 2.11. Lipidomics

Lipidomic analysis of mouse liver tissue was performed using reverse-phase liquid chromatography–tandem mass spectrometry (RP-LC–MS/MS) on a SCIEX ExionLC system coupled to a SCIEX X500R QTOF mass spectrometer (SCIEX, Framingham, MA, USA). Separations employed a Kinetex C18 column (2.1 mm × 100 mm, 2.6 μm; 40 °C) at 0.30 mL/min, with mobile phase A consisting of acetonitrile/methanol/water (1:1:1, *v*/*v*/*v*) and mobile phase B of isopropanol, both supplemented with 5 mM ammonium acetate. A 1 μL sample was injected and analyzed in two complementary modes: positive electrospray ionization (ESI+; 5.5 kV spray voltage) and negative ESI (ESI−; 4.5 kV spray voltage). Key mass spectrometry parameters included a 550 °C source temperature, 35 psi curtain gas, 80 V declustering potential, and collision energy at 35 ± 15 V with dynamic background subtraction. The data were acquired in information-dependent acquisition (IDA) mode, with TOF MS scans (*m*/*z* 100–1500; 200 ms accumulation) triggering MS/MS scans (*m*/*z* 50–1500; 50 ms accumulation). Raw data were processed in MS-DIAL (v5.1) for lipid identification via exact mass, retention time, and MS/MS spectral matching against reference libraries. Identifications were validated through manual MS/MS spectral inspection using PeakView (v2.2, SCIEX, Framingham, MA, USA), followed by peak area quantification in MultiQuant (v3.0, SCIEX) to determine relative lipid abundance. Peak intensities were log-transformed and autoscaled (mean-centered/unit variance scaled) in MetaboAnalyst 6.0. Quality control was enforced by excluding lipid features with >30% relative standard deviation (RSD) in QC samples to ensure data reliability.

### 2.12. Network Pharmacology

Network pharmacology was employed to predict between DIM and MASLD. Briefly, DIM-associated genes were identified by querying ‘diindolylmethane’ in the Similarity Ensemble Approach (SEA) and GeneCards databases. MASLD-related genes were retrieved from GeneCards using ‘nonalcoholic fatty liver disease’ as the search term. Overlapping genes between DIM and MASLD targets were identified via Venn analysis [21]. Protein–protein interactions (PPIs) among these overlapping genes were then analyzed using the STRING database (v 12.0, Search Tool for Retrieval of Interacting Genes/Proteins) at a high-confidence interaction score threshold (≥0.9). The resulting PPI network was imported into Cytoscape (v3.10.3), where disconnected nodes were removed, and topological features of the network were subsequently characterized.

### 2.13. Molecular Docking

The three-dimensional structure of DIM was retrieved from PubChem (https://pubchem.ncbi.nlm.nih.gov). Structures of the AMPK α1 and AMPK α2 subunits were similarly obtained from PDB website (https://www.rcsb.org/). Both AMPK subunits served as receptor proteins, with DIM acting as the ligand. All structures were imported into the Molecular Operating Environment (MOE) software (version 2022.02, Chemical Computing Group Inc., Montreal, QC, Canada). Following energy minimization and structural preprocessing of both receptors and ligand, molecular docking simulations were performed using standardized MOE protocols. The optimal docking conformations, selected based on binding energy and interaction stability, were retained for analysis. Binding modes and intermolecular interactions between DIM and the ligand-binding domains of AMPK α1 and AMPK α2 were analyzed, and the most stable complexes were visualized.

### 2.14. Statistical Analysis

Data are expressed as mean ± standard deviation (SD). Statistical analyses were performed using SPSS 26.0 (IBM Corp., Armonk, NY, USA). For multi-group comparisons, one-way ANOVA was applied to normally distributed data. Post hoc analyses utilized either the LSD test (for homogeneous variances) or Dunnett’s T3 test (for heterogeneous variances), with variance homogeneity verified by Levene’s test.

## 3. Results

### 3.1. DIM Ameliorated Metabolic Disturbance Induced by HFD in WT Mice

**Figure 1:**
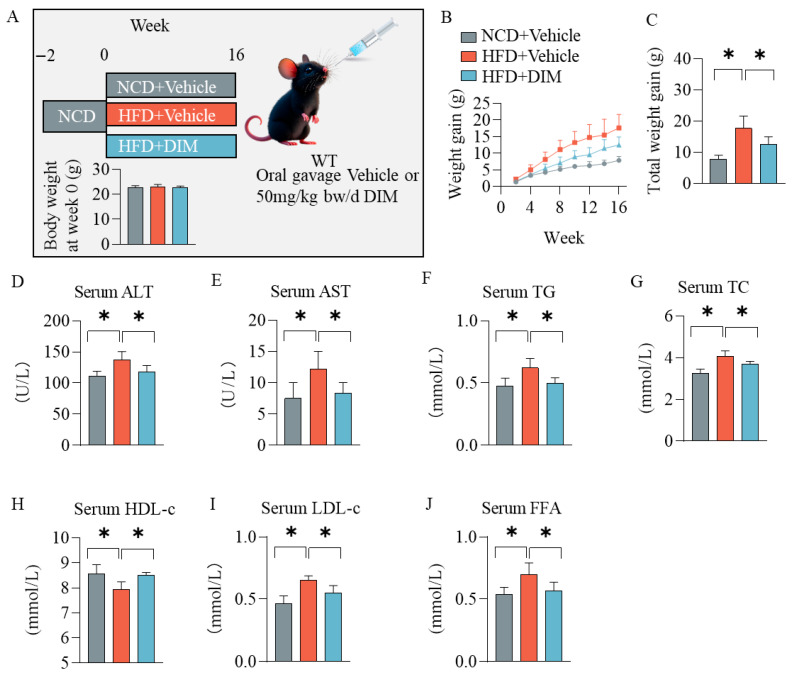
Effects of DIM on serum biochemical parameters. (**A**) Schematic diagram of the experimental design. (**B**,**C**) Body weight of mice was collected during the experiment. (**D**–**J**) Serum ALT, AST, TG, TC, HDL-c, LDL-c, and FFA were detected. The data are presented as mean ± SD (*n* ≥ 3) from at least three independent biological replicates. * *p* < 0.05.

The experimental procedure for the in vivo study is shown in Figure 1A. There was no significant difference in initial weight among the three groups (Figure 1A). After 16 weeks of treatment, mice receiving the HFD demonstrated significantly elevated body weight gain relative to those fed the NCD (Figure 1B,C). DIM significantly prevented the body weight gain induced by HFD (Figure 1B,C). To validate HFD efficacy, serum biochemical parameters related to lipid metabolism and liver function were assessed. HFD feeding induced significant dyslipidemia, characterized by elevated serum TG, TC, LDL-c, and FFA, alongside reduced HDL-c, and increased hepatic damage markers (ALT, AST) relative to NCD feeding (Figure 1D–J). DIM treatment counteracted the HFD-induced effects, significantly reversing the elevated serum levels of ALT, AST, TG, TC, LDL-c, and FFA, and restoring HDL-c levels (Figure 1D–J).

### 3.2. DIM Prevented Hepatic Lipid Deposition and Oxidative Stress in WT Mice Fed with HFD

**Figure 2:**
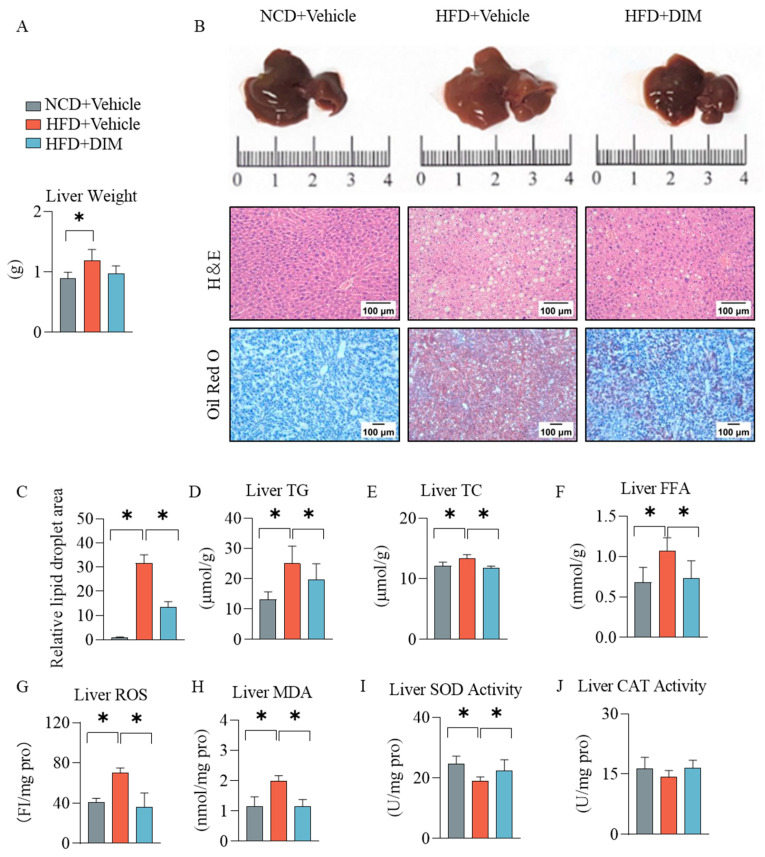
Effects of DIM on hepatic lipid deposition and oxidative stress. (**A**) Liver weights of mice. (**B**) The appearance of livers and H&E and Oil Red O staining of liver tissue. (**C**) Quantification of Oil Red O staining. (**D**–**J**) Hepatic levels of TG, TC, FFA, ROS, MDA, SOD activity, and CAT activity were quantification. The data are presented as mean ± SD (*n* ≥ 3) from at least three independent biological replicates. * *p* < 0.05.

Livers from HFD-fed mice displayed pathological enlargement, grayish-yellow discoloration, and a greasy appearance characteristic of hepatic lipid accumulation. DIM-treated mice exhibited livers with normal reddish-brown pigmentation, smooth surfaces, and uniform texture (Figure 2A,B). A detailed histopathological examination of DIM’s effects was performed using H&E and Oil Red O staining of liver tissues. H&E and Oil Red O staining revealed that HFD induced intracytoplasmic accumulation of variably sized lipid droplets in hepatocytes, whereas DIM treatment significantly attenuated hepatic lipid deposition (Figure 2B,C). DIM significantly attenuated HFD-induced elevations in hepatic TG, TC, and FFA content. Then we assessed whether DIM counteracted oxidative stress in liver. Compared to NCD group, HFD group significantly increased hepatic ROS and MDA levels while decreasing SOD activity; however, hepatic CAT activity remained unaffected (Figure 2G–I). DIM treatment significantly counteracted HFD-driven elevations in hepatic oxidative stress markers (ROS and MDA) while restoring SOD activity (Figure 2G–I).

### 3.3. DIM Mitigated Hepatic Lipid Deposition and Oxidative Stress in Genetically Obese (ob/ob) Mice

**Figure 3:**
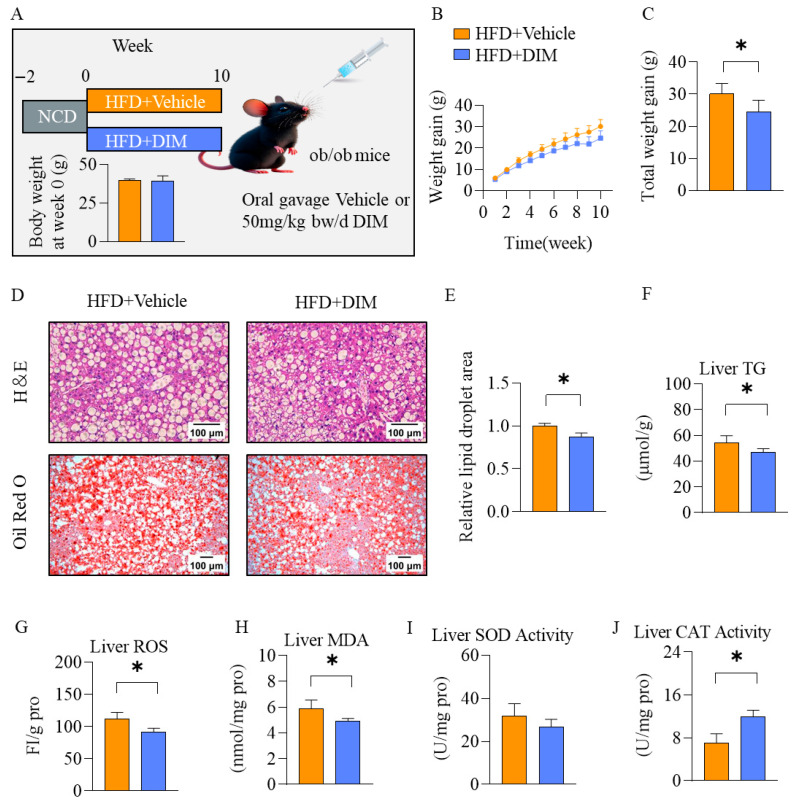
The effects of DIM on ob/ob mice fed with HFD. (**A**) Schematic diagram of the experimental design. (**B**,**C**) Body weight gain of mice. (**D**) H&E and Oil Red O staining of liver tissue. (**E**) Quantification of Oil Red O staining. (**F**–**J**) Hepatic levels of TG, ROS, MDA, SOD activity, and CAT activity. The data are presented as mean ± SD (*n* ≥ 3) from at least three independent biological replicates. * *p* < 0.05.

Given the heterogeneous nature of MASLD across its disease spectrum, the ob/ob mouse model fed a high-fat diet (HFD) was employed to study whether DIM ameliorated MASLD (Figure 3A). There were no significant differences in body weights at week 0 (Figure 3A). After 10-week treatment, DIM dramatically prevented the body weight gain (Figure 3B,C). H&E and Oil Red O staining demonstrated pronounced HFD-induced lipid vacuolization throughout hepatic lobules in ob/ob mice, an effect substantially alleviated by DIM treatment (Figure 3D,E). Biochemical analysis demonstrated that DIM reduced hepatic TG content, ROS and MDA levels, and enhanced CAT activity, while exerting no significant effect on SOD activity in the liver (Figure 3F–J).

### 3.4. DIM Reshaped the Hepatic Lipid Profile in Mice

**Figure 4:**
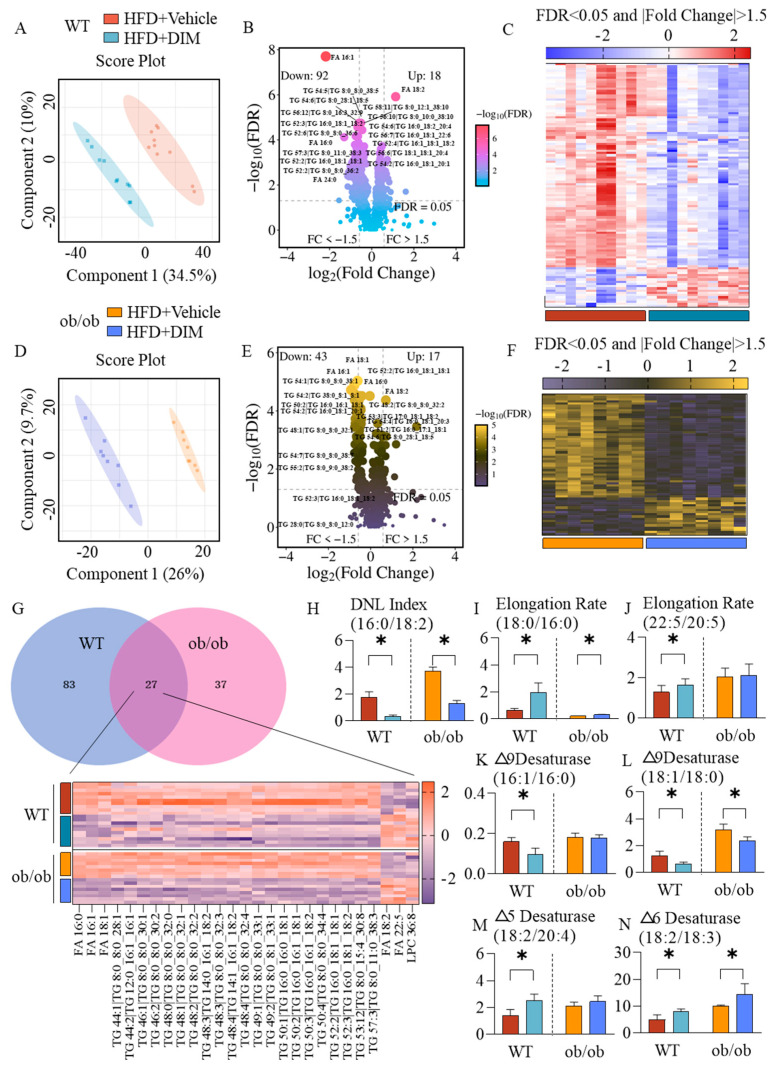
Lipidomics in mouse liver. (**A**) Score plot of the PLS-DA analysis in WT mice. (**B**,**C**) Volcano plot and heatmap of lipid species differed between two groups in WT mice, FDR < 0.05 and |fold change| > 1.5. (**D**) Score plot of the PLS-DA analysis in ob/ob mice. (**E**,**F**) Volcano plot and heatmap of lipid species differed between two groups in ob/ob mice, FDR < 0.05 and |fold change| > 1.5. (**G**) Venn diagram and heatmap of targets shared by WT and ob/ob mice. (**H**–**N**) The hepatic DNL index, elongation rate, △9, △5, △6-desaturase activity in WT and ob/ob mice. The data are presented as mean ± SD (*n* ≥ 3) from at least three independent biological replicates. * *p* < 0.05.

In order to observe the DIM-induced changes in lipid metabolism, untarget lipidomic was used to uncover the hepatic lipid profile. In WT and ob/ob mice, PLS-DA analysis mostly segregated the mice treated with vehicle or DIM (Figure 4A,D). DIM modulated lipid species abundance differentially between WT and ob/ob mice: it elevated 18 and suppressed 92 species in WT mice, versus increasing 17 and reducing 43 in ob/ob mice (FDR < 0.05 and |fold change| > 1.5, Figure 4B,C,E,F). The Venn diagram reveals a core set of 27 lipid species exhibiting significant alterations in both wild-type (WT) and ob/ob mouse livers by DIM (Figure 4G). DIM reduced the lipogenic index (FA 16:0/FA 18:2 ratio), a biomarker of de novo lipogenesis (DNL) in HFD-fed animals, in both WT and ob/ob mice (Figure 4H). DIM significantly increased fatty acid elongation indices in both genotypes: the FA 18:0/16:0 ratio was elevated universally, while the very long-chain elongation index (FA 22:5/20:5) increased specifically in WT mice (Figure 4I,J). DIM bidirectionally modulated desaturase activities: while inhibiting △9-desaturase (reduced FA 16:1/16:0 in WT; decreased FA 18:1/18:0 in both genotypes), it activated △5-desaturase (elevated FA 20:4/18:2 in WT) and △6-desaturase (increased FA 18:2/18:3 in both genotypes) (Figure 4K–N).

### 3.5. DIM Modulates Hepatic Signaling Pathways Involved in Lipid Metabolism

**Figure 5:**
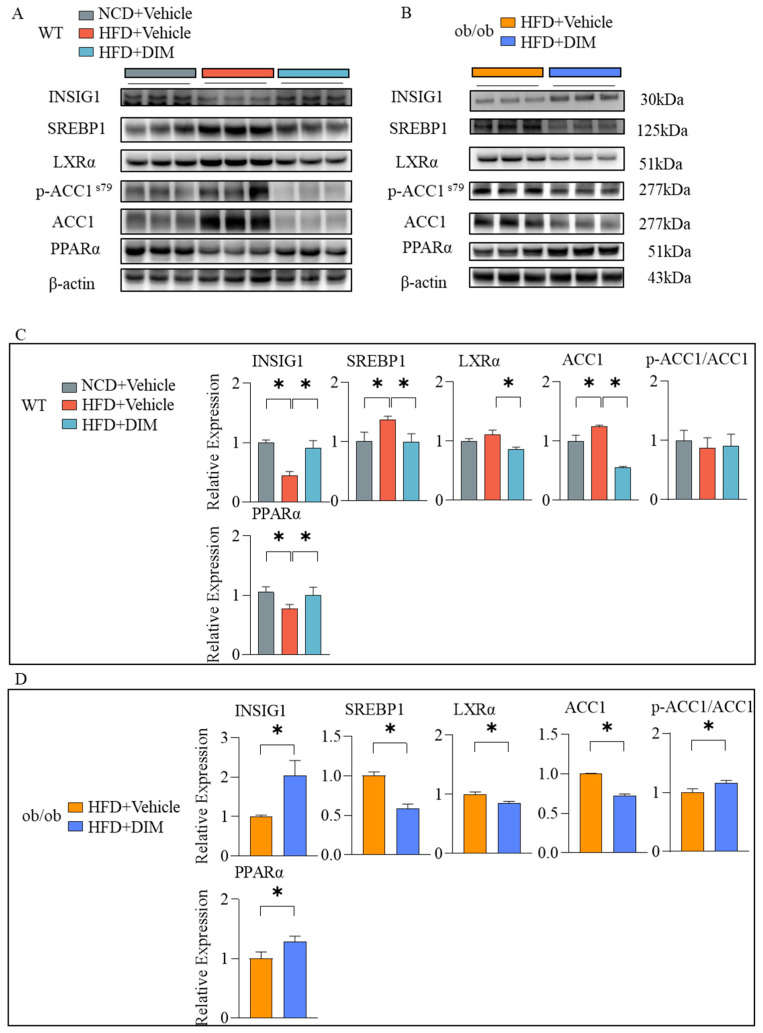
Effects of DIM on lipogenesis and fatty acid oxidation. (**A**–**D**) Protein levels of INSIG1, SREBP1, LXRα, ACC1, p-ACC1, and PPARαin in mouse liver. The data are presented as mean ± SD (*n* ≥ 3) from at least three independent biological replicates. * *p* < 0.05.

In light of significant changes in hepatic lipid accumulation, we assessed the expression of genes governing lipogenesis and fatty acid oxidation. Previous studies indicate that Insulin-induced gene 1 (INSIG1) binds the Sterol-regulatory element binding protein (SREBP) precursor and SREBP cleavage-activating protein (SCAP) on the endoplasmic reticulum membrane, retaining the SREBP-SCAP complex to inhibit proteolytic activation. This prevents nuclear translocation of SREBP1 and subsequent transcription of genes involved in cholesterol and fatty acid synthesis [22]. In WT and ob/ob mice, DIM increased the levels of INSIG1 and decreased the level of SREBP1 (Figure 5A–D). Then we test the downstream key fatty acid synthase Acetyl-CoA Carboxylase 1 (ACC1). In WT and ob/ob mice, DIM downregulated the levels of ACC1. In ob/ob mice, DIM increased the inhibitory phosphorylation in ob/ob mice (Figure 5A–D). Moreover, liver X receptors (LXRs) are the upstream regulators of SREBP1. DIM reduced the level of LXRα (Figure 5A–D). Subsequently, we measured protein levels of Peroxisome Proliferator-Activated Receptor α (PPARα; a master transcriptional controller of β-oxidation). The DIM group markedly enhanced the protein levels of PPARαin WT and ob/ob mice relative to HFD group (Figure 5A–D).

### 3.6. DIM Triggered the Hepatic Phosphorylation of AMPKα in Mice

**Figure 6:**
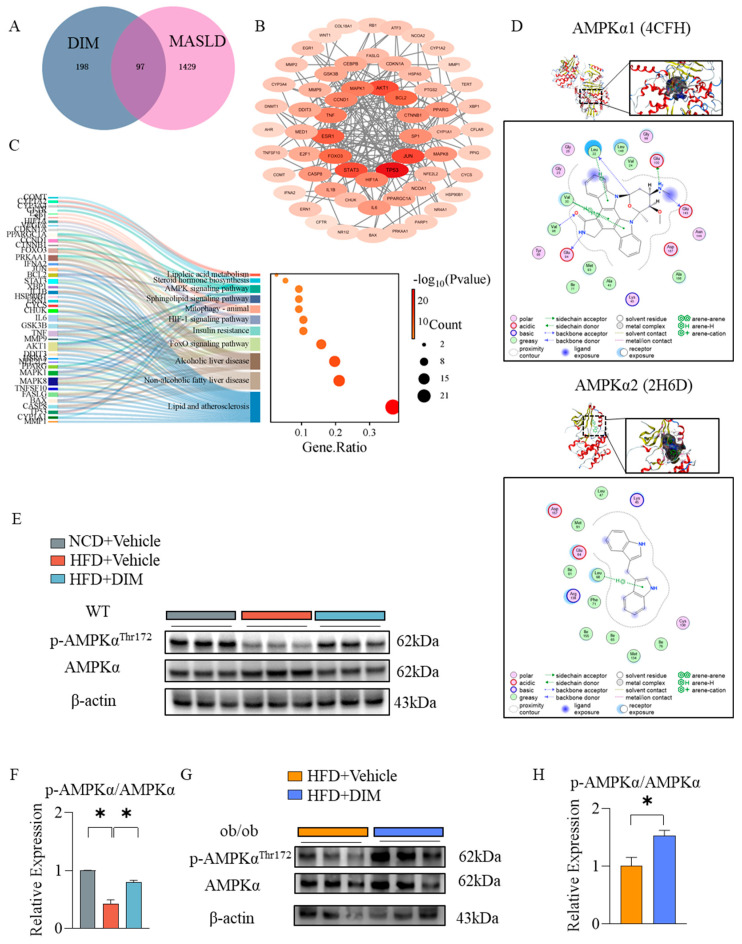
Network pharmacology and molecular docking predicted possible mechanisms of DIM for MASLD. (**A**) Venn diagram of targets shared by DIM and MASLD. (**B**) PPI network of common targets. (**C**) KEGG analysis of common genes. (**D**) Molecular docking of DIM and AMPKα1 or AMPKα2. (**E**–**H**) Hepatic protein levels in WT or ob/ob mice. The data are presented as mean ± SD (*n* ≥ 3) from at least three independent biological replicates. * *p* < 0.05.

Network pharmacology and molecular docking are extensively employed in drug discovery research. This study identified 295 putative therapeutic targets of DIM and 1526 genes associated with MASLD. Cross-referencing these targets yielded 97 overlapping targets relevant to both the drug and the disease (Figure 6A). The PPI network analysis revealed high combined interaction scores for genes implicated in tumor progression, inflammatory responses, and glucose/lipid metabolism (Figure 6B). From KEGG enrichment analysis of glycolipid metabolism-related pathways, the “Lipid and atherosclerosis”, “Non-alcoholic fatty liver disease”, “Alcoholic liver disease”, “FoxO signaling pathway”, “Insulin resistance”, “HIF-1 signaling pathway”, “Mitophagy”, “Sphingolipid signaling pathway”, “AMPK signaling pathway”, “Steroid hormone biosynthesis”, and “Linoleic acid metabolism” emerged as significantly enriched (Figure 6C). These results indicated that DIM regulated oxidative stress, lipid profile and insulin sensitivity in MASLD. Molecular docking analysis revealed that DIM forms hydrogen bonds with Glu100 of Adenosine 5‘-monophosphate (AMP)-activated protein kinase (AMPK) α1 and Leu68 of AMPKα2, with calculated binding energies of −9.2 kcal/mol and −27.3 kcal/mol, respectively (Figure 6D). Then we test the activity of AMPK in WT and ob/ob mice. HFD suppressed AMPKα phosphorylation in WT mice, whereas DIM administration restored it in WT and ob/ob mice (Figure 6E–H).

### 3.7. DIM Mitigated Cellular Lipid Deposition and Oxidative Stress Through AMPKα In Vitro

**Figure 7:**
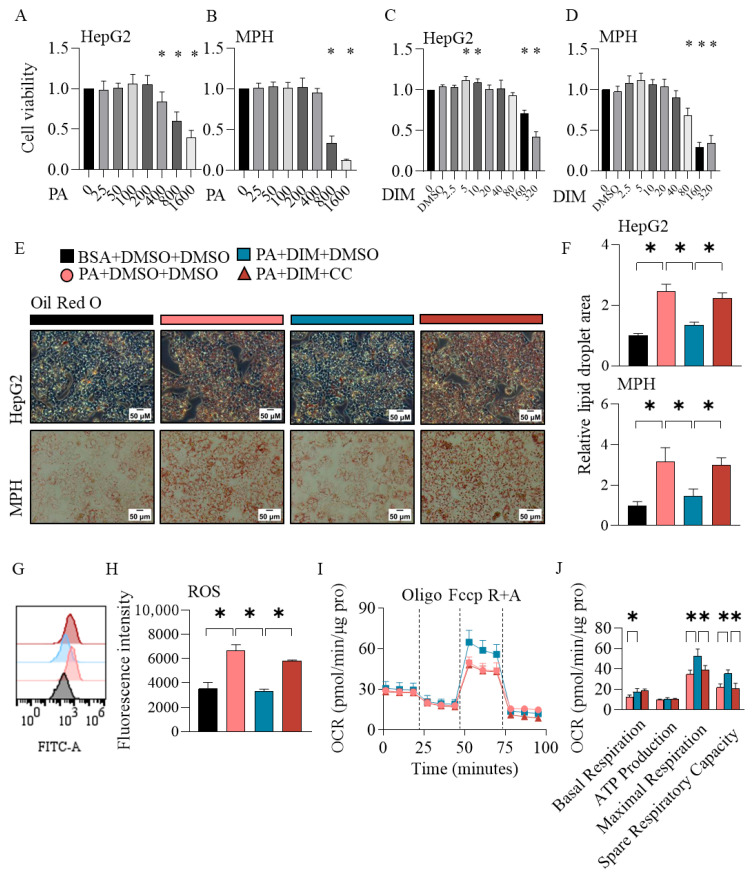
The effects of DIM on cellular lipid deposition and oxidative stress in vitro. (**A**–**D**) The results of CCK8 in HepG2 and MPH. (**E**,**F**) Oil Red O staining and its quantification. (**G**,**H**) Cellular ROS content of MPH. (**I**,**J**) Seahorse assay on MPH. (**E**–**J**) shared the same legend. The data are presented as mean ± SD (*n* ≥ 3) from at least three independent biological replicates. * *p* < 0.05.

HepG2 cells and MPH were utilized to assess whether DIM improves cellular lipid metabolism by activating AMPKα. CCK-8 assays demonstrated dose-dependent PA cytotoxicity: significant viability reductions occurred at 400–1600 μM in HepG2 cells, while only 800–1600 μM impaired cell viability in MPH (Figure 7A,B). Therefore, 300 μM PA was used for cellular lipid deposition model establishment. Based on dose–response viability data (Figure 7C,D), a non-toxic DIM concentration (40 μM) was utilized to investigate AMPK-mediated effects. Oil Red O staining revealed that 40 μM DIM significantly attenuated PA-induced lipid accumulation in both HepG2 cells and MPH (Figure 7E,F). This protective effect was abolished by pretreatment with the AMPK-specific inhibitor CC, confirming AMPK-dependence of DIM’s lipid-lowering action (Figure 7E,F). In order to test whether DIM ameliorated oxidative stress through AMPKα, the cellular ROS of MPH was stained. DIM repressed the elevation of ROS induced by PA, while CC markedly increased cellular ROS content (Figure 7G,H). Then, we tested the oxygen consumption rate in MPH. DIM significantly enhanced basal respiration, maximal respiration, and spare respiratory capacity in MPH. Conversely, pretreatment with CC selectively attenuated DIM-induced increases in maximal respiration and spare respiratory capacity (Figure 7I,J).

### 3.8. DIM Stimulated Lipogenesis and Fatty Acid Oxidation Through AMPK

**Figure 8:**
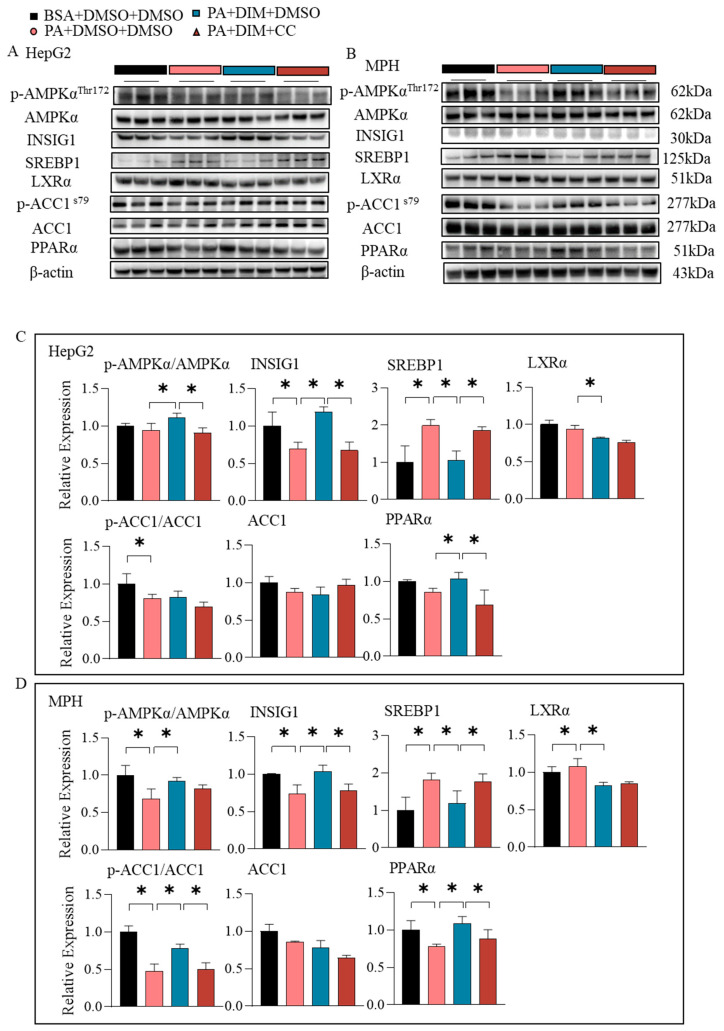
The effects of DIM on lipogenesis and fatty acid oxidation in vitro. (**A**–**D**) The protein levels of p-AMPK, INSIG1, SREBP1, LXRα, ACC1, p-ACC1, and PPARα in HepG2 cells and MPH. The data are presented as mean ± SD (*n* ≥ 3) from at least three independent biological replicates. * *p* < 0.05.

Finally, the expression of genes governing lipogenesis and fatty acid oxidation were detected. In both HepG2 cells and MPH, PA significantly downregulated protein expression of p-AMPKα, INSIG1, and PPARα, while concurrently upregulating SREBP1 protein levels (Figure 8A–D). The ability of DIM to counteract PA-induced dysregulation of metabolic regulators was abrogated by AMPK inhibition with CC, demonstrating that DIM’s benefits require AMPK activation (Figure 8A–D).

## 4. Discussion

The present study found that DIM counteracted diet or genetic-induced hepatic lipid deposition by activating AMPKα in liver. Administration of DIM promoted AMPKα phosphorylation, concurrently reducing the lipogenic transcription factor SREBP1 while elevating the fatty acid-oxidizing transcription factor PPARα.

DIM, a bioactive lipophilic compound abundant in dietary Brassica vegetables (e.g., kale, broccoli, cauliflower), forms endogenously from Indole-3-carbinol (I3C) [23]. Within the acidic gastric environment, I3C undergoes self-condensation, forming a mixture of condensation products, among which DIM predominates [23]. Supplementation with 0.1% I3C in HFD ameliorated hepatic steatosis, achieved through marked suppression of SREBP1-regulated lipogenic enzyme expression, and subsequent attenuation of hepatic lipid accumulation [24]. Therefore, DIM might have similar beneficial effects on MASLD. Human study indicated that single doses of DIM under 300 mg/day are well tolerated by healthy subjects (using standard body surface area conversion [14], the human-equivalent dose (based on a 60 kg body weight) translates to a mouse dose of 61.67 mg/kg bw/day) [15]. Moreover, DIM was administered to mice via oral gavage at 250 mg/kg with no observed toxicity and exhibited broad tissue distribution [13]. Therefore, we did not observe the adverse effects of 50 mg/kg bw/day DIM in mice.

The pathogenesis of MASLD is best explained by the “multiple-hit” hypothesis, which encompasses the synergistic interplay of diverse factors including diet, genetic predispositions, oxidative stress, insulin resistance, and inflammation in driving disease initiation and progression [25]. Hepatic lipid accumulation driven by this process induces hepatocellular injury, resulting in further TG deposition and elevated AST/ALT levels. In present study, administration of 50 mg/kg BW/day DIM attenuated hepatic lipid accumulation, peroxidation and hepatocellular damage in MASLD models driven by both dietary (HFD) and genetic (obesity) factors.

AMPK, a heterotrimeric kinase with α/β/γ subunits, regulates cellular metabolism through phosphorylation-dependent activation at multiple subunit sites [26]. Thr172 phosphorylation activates the AMPKα-subunit, thereby modulating hepatic glycolipid metabolism via improved insulin sensitivity, suppressed lipogenesis, and enhanced fatty acid oxidation [27]. Using network pharmacology, we predicted DIM’s functional mechanisms in MASLD. The analysis identified 97 targets modulating MASLD pathogenesis, with the AMPK pathway demonstrating the strong association with hepatic lipid homeostasis. Molecular docking demonstrated binding of DIM to both AMPKα1 and AMPKα2, with superior binding affinity for AMPKα2. Furthermore, DIM induced AMPKα phosphorylation both in vivo and in vitro. Therefore, DIM might induced the Thr172 phosphorylation of AMPK by binding to its α unit. Crucially, AMPK-specific inhibition abrogated DIM-mediated attenuation of hepatic steatosis and metabolic dysregulation. In adipose tissue, Lee et al. demonstrated that DIM prevented adipogenesis by modulating AMPK activity [28]. This finding supports our hypothesis that DIM activates AMPK. Moreover, in our previous research, we demonstrated that DIM regulates hepatic lipid accumulation through the aryl hydrocarbon receptor (AhR)-MAPK signaling pathway in hepatocytes [29]. This finding is further supported by network pharmacology analysis revealing significant enrichment of the MAPK pathway. Building on this evidence and our current results, we propose that DIM ameliorates hepatic lipid metabolism not only via MAPK signaling but also through activation of the AMPK pathway in hepatocytes. Collectively, these findings establish AMPK activation as necessary for DIM-mediated regulation of hepatic lipid homeostasis.

The lipogenesis and fatty acid oxidation are important for hepatic lipid metabolism. SREBP1, a key member of the sterol regulatory element-binding protein family, functions as a master transcriptional regulator of lipogenesis. Primarily expressed in hepatocytes and adipocytes, it governs genes involved in triglyceride synthesis, fatty acid metabolism, and glucose homeostasis [30]. AMPK activation induced Ser372 phosphorylation of SREBP1c, which targets the transcription factor for ubiquitin-proteasomal degradation [31]. Therefore, we observed the downregulation of SREBP1 by DIM in vivo and in vitro. DIM significantly suppressed lipogenesis, as evidenced by reduced ACC1 protein levels, decreased palmitic acid (FA 16:0) content, and diminished DNL activity. ACC1, a key transcriptional target of SREBP1, catalyzes the rate-limiting carboxylation step in de novo palmitic acid synthesis [32]. Excess palmitic acid production is recognized as a key driver of MASLD pathogenesis, promoting hepatic TG accumulation, insulin resistance, and inflammation through lipotoxic mechanisms [33]. Therefore, DIM regulated hepatic lipogenesis by AMPK-SREBP-ACC1 signaling pathway. INSIG1, an endoplasmic reticulum (ER) membrane-resident protein, functions as a gatekeeper in the SREBP regulatory pathway. AMPK interacts with and phosphorylates INSIG1; this post-translational modification disrupts INSIG1’s association with the E3 ubiquitin ligase gp78, thereby suppressing ubiquitination and degradation of INSIG1 [34]. In the present study, DIM increased the hepatic INSIG1 levels. Therefore, the activation of AMPKα by DIM repressed SREBP1 through multiple signaling pathway, ultimately reducing the downstream fatty acid synthesis.

PPARα, a ligand-inducible nuclear receptor, transcriptionally regulates genes governing fatty acid oxidation, elongation/desaturation, and TG cycling [35]. Prior research demonstrates that AMPK phosphorylates peroxisome-proliferator-activated receptor gamma coactivator 1 alpha (PGC-1α), which in turn activates PPARα [36]. Given the presence of peroxisome proliferator response elements (PPRE) motifs in promoters of PPARα [37], we propose that DIM-mediated AMPK activation enhances PPARα transcriptional activity, potentially elevating its expression. Moreover, we observed the downregulation of DNL rate and hepatic level of FFA by DIM in mice. This inhibition reduces malonyl-CoA production, thereby promoting fatty acid oxidation (FAO) [38]. Network pharmacology revealed significant enrichment in linoleic acid metabolism pathways, while lipidomics demonstrated DIM-induced downregulation of Δ9-desaturase activity, coordinated upregulation of fatty acid elongation, and enhanced Δ5/Δ6-desaturase activities. The activation of PPARα by DIM might be responsible for the fatty acid elongation and desaturation. Moreover, these results indicate DIM redirects hepatic metabolic flux toward polyunsaturated fatty acid (PUFA) biosynthesis. Although β-oxidation preferentially utilizes saturated fatty acids or Δ2-trans-enoyl-CoA intermediates, naturally occurring cis-configured double bonds impede this process [39]. Paradoxically, PUFA serve as potent PPARα ligands that transcriptionally upregulate β-oxidation machinery, ultimately enhancing hepatic fatty acid catabolism [40]. Consequently, DIM reduced hepatic DNL and FFA accumulation while increasing OCR, reflecting elevated mitochondrial FAO.

This study employs a single-target approach, integrating both in vitro (cell-based) and in vivo (animal) experimental models. A key limitation of the current work lies in the absence of human population data to validate its precision and generalizability. To address this, a robust clinical translation framework should be developed to solidify its safety profile in clinical applications. Furthermore, while DIM exhibits predominant hepatic accumulation, systemic exposure to other organs following administration is inevitable. Subsequent investigations should therefore prioritize exploring its multi-target synergistic effects to provide a more comprehensive understanding of its biological activity.

In summary, DIM ameliorates MASLD through dual AMPK-dependent pathways: (1) suppression of hepatic de novo lipogenesis via AMPK-mediated SREBP1 downregulation, and (2) enhancement of fatty acid β-oxidation through PPARα transactivation.

## Data Availability

The data is contained within the article and the Supplementary Materials.

## Supplementary Materials

The following supporting information can be downloaded at https://www.mdpi.com/article/10.3390/antiox14091093/s1: Table S1: Antibodies Used.

## Abbreviations

The following abbreviations are used in this manuscript:

MASLD	Metabolic dysfunction-associated steatotic liver disease
DNL	De novo lipogenesis
VLDL	Very low-density lipoprotein
TG	Triglyceride
HFD	High-fat diet
DIM	3,3′-Diindolylmethane
WT	Wild-type
SPF	Specific pathogen-free
CMC-Na	Sodium carboxymethyl cellulose
NCD	Normal chow diet
FFA	Free fatty acids
TC	Total cholesterol
ROS	Reactive oxygen species
MDA	Malondialdehyde
CAT	Catalase
SOD	Superoxide dismutase
LDL-c	Low density lipoprotein-cholesterol
HDL-c	High density lipoprotein-cholesterol
ALT	Alanine aminotransferase
AST	Aspartate aminotransferase
H&E	Hematoxylin and eosin
PVDF	Polyvinylidene difluoride
BSA	Bovine serum albumin
MPH	Mouse primary hepatocytes
FBS	Fetal bovine serum
PA	Palmitate
CC	Compound C
OCR	Oxygen consumption rate
RP-LC–MS/MS	Reverse-phase liquid chromatography–tandem mass spectrometry
IDA	Information-dependent acquisition
SD	Standard deviation
INSIGG1	Insulin-induced gene 1
SREBP	Sterol-regulatory element binding protein
ACC1	Acetyl-CoA Carboxylase 1
LXR	Liver X receptors
PPARα	Peroxisome Proliferator-Activated Receptor α
AMPK	Adenosine 5‘-monophosphate (AMP)-activated protein kinase
I3C	Indole-3-carbinol
ER	Endoplasmic reticulum
PGC-1α	Peroxisome-proliferator-activated receptor gamma coactivator 1 alpha
PPRE	Peroxisome proliferator response elements
PUFA	Polyunsaturated fatty acid
