# Supplementary material for: Integrated Lipidomics and Network Pharmacology Reveal the AMPK-Mediated Therapeutic Mechanism of 3,3′-Diindolylmethane in Hepatic Lipid Metabolism
Source: Antioxidants (Basel). 2025 Sep 7;14(9):1093. doi: 10.3390/antiox14091093 (PMC12466802; doi:10.3390/antiox14091093)
Supplement: Supplementary file 1 [file antioxidants-14-01093-s001.zip › antioxidants-3795351-supplementary.pdf]

Table S1 Antibodies Used

Antibodies	Dilution	Corporation	Identifier
p-AMPKα (Thr172) Rabbit Monoclonal Antibody primary antibody	1:1000	Cell Signaling Technology	2535
AMPKα Rabbit Polyclonal antibody	1:1000	Cell Signaling Technology	2532
INSIG-1Mouse Monoclonal Antibody	1:1000	Santa Cruz	sc-390504
SREBP-1 Mouse Monoclonal Antibody	1:500	Santa Cruz	sc-13551
ACC1 Rabbit Monoclonal Antibody	1:1000	Abclonal	A19627
p-ACC1(s79) Rabbit Monoclonal Antibody	1:500	Abclonal	AP0298
ACOX1 Rabbit Monoclonal Antibody	1:1000	Abclonal	A21217
LXRα Rabbit Monoclonal Antibody	1:1000	Abclonal	A3974
PPARα Rabbit Polyclonal antibody	1:1000	Bioss	bs-3614R
β-actin Mouse Monoclonal Antibody	1:5000	Rayantibody	RM2001
Goat Anti-Mouse IgG (HRP)	1:5000	Rayantibody	RM3001
Goat Anti-Rabbit IgG (HRP)	1:5000	Rayantibody	RM3002
